# Benefits of local tumor excision and pharyngectomy on the survival of nasopharyngeal carcinoma patients: a retrospective observational study based on SEER database

**DOI:** 10.1186/s12967-017-1204-x

**Published:** 2017-05-30

**Authors:** Jian Sun, Zhongying Huang, Zheyu Hu, Rui Sun

**Affiliations:** 1 Department of Clinical Research, Sun Yat-sen University Cancer Center, State Key Laboratory of Oncology in South China, Collaborative Innovation Center for Cancer Medicine, No. 651 Dongfeng Road, East, Guangzhou, 510060 Guangdong People’s Republic of China; 2 Department of Clinical Research and Teaching, First Hospital of Changsha City, No. 311 Yinpan Road, East, Changsha, 410005 People’s Republic of China; 3 Department of Nasopharyngeal Carcinoma, Sun Yat-sen University Cancer Center, State Key Laboratory of Oncology in South China, Collaborative Innovation Center for Cancer Medicine, No. 651, Dongfeng Road East, Guangzhou, 510060 Guangdong People’s Republic of China

**Keywords:** Nasopharyngeal carcinoma (NPC), Epidemiology and End results (SEER) database, Propensity score, Local tumor excision, Pharyngectomy

## Abstract

**Background:**

There is ongoing debate about surgery of primary site in nasopharyngeal carcinoma patients.

**Methods:**

3919 patients with nasopharyngeal carcinoma identified in the SEER registry between 2004 and 2013. The benefit of surgery of primary nasopharynx tumor site on overall and cancer-specific survival was assessed by risk-adjusted multivariate Cox proportional hazard regression and propensity score matching modeling.

**Results:**

Surgery was marginally associated with better overall survival (hazard ratio (HR) = 0.816, 95% CI 0.656–1.015, p = 0.07) and cancer-specific survival (HR = 0.749, 95% CI 0.552–1.018, p = 0.06) in the propensity score model. Among 398 cases who underwent primary site surgery, 282 (70.85%) received local tumor excision and 79 (20.31%) received pharyngectomy. Local tumor excision and pharyngectomy had almost the same effect on survival in propensity score matching analysis. The benefit was significant in subgroups of white, age <60 year, and patients with T3, N1, M0, AJCC stage III, or moderately differentiated tumors. Further survival analysis showed surgery to promote survival in both radiotherapy and non-radiotherapy patients.

**Conclusion:**

This is the first population-based analysis using propensity score model to provide evidence of a positive impact of surgery on survival in nasopharyngeal carcinoma. Moreover, surgery demonstrated the significant benefit in subgroups of patients with specific clinical characteristics.

**Electronic supplementary material:**

The online version of this article (doi:10.1186/s12967-017-1204-x) contains supplementary material, which is available to authorized users.

## Background

Nasopharyngeal carcinoma is a rare type of head and neck cancer. It is uncommon in countries other than Asia [1]. In the US, nasopharyngeal carcinoma has been seen in Asian Americans, African Americans, Hispanics and white. Based on US National Cancer Institute’s (NCI) Surveillance, Epidemiology, and End Results (SEER) registry data 1988–2010, African American and Asian patients with nasopharyngeal carcinoma have better disease-specific survival when compared to Caucasian patients [2].

Nasopharyngeal carcinoma has a high propensity to metastasize to distant sites, and poses a significant risk for isolated local recurrences after radiation for locally advanced disease [3, 4]. Due to treatment failure, it causes 65,000 deaths globally in 2010 [5]. According to American Joint Committee on Cancer (AJCC) TNM staging for nasopharyngeal carcinoma (7th ed., 2010), stage is accepted as prognostically important [6]. Relative 5-year survival rates for stage I, II, III and IV patients were 72, 64, 62 and 38%, respectively.

According to World Health Organization (WHO) classification, nasopharyngeal carcinoma histology and differentiation subtypes include differentiated keratinizing squamous cell carcinoma (K-NPSCC), differentiated non-keratinizing squamous cell carcinoma (NK-NPSCC) and undifferentiated carcinoma. Based on SEER registry data till 2010, NK-NPSCC showed a better prognosis than keratinizing-NPSCC [7], because keratinizing squamous cell cancers have a higher incidence of deaths from uncontrolled primary tumors and nodal metastases [8].

According to National Comprehensive Cancer Network (NCCN) guidelines, patients with T1, N0, M0 Nasopharyngeal carcinoma may be treated with definitive radiotherapy (RT) alone [9]. RT plus chemotherapy is recommended for T1, N1–N2 or T2–T4, any N lesions patients [10, 11]. For metastatic disease, platinum-based combination chemotherapy regimen or concurrent chemotherapy/RT is recommended [10, 12]. Advances in skull base surgery make possible the effective control of primary recurrence of nasopharyngeal carcinoma [13–15]. Radical neck dissection is safe and effective in the treatment of the neck failure [15], but patients with age >50, stage N3, or LN >6 cm have poor prognosis [16].

The SEER program of NCI is a population-based cancer registry covering approximately 30% of the population in the United States. This database is the largest publicly available and authoritative information source on cancer incidence and survival. Using this reliable and large-scale research dataset, we were able to statistically analyze the survival outcomes for patients with nasopharyngeal carcinoma.

The objective of this study was to evaluate the surgery treatment on survival of patients diagnosed with primary nasopharyngeal carcinoma using the case information extracted from the SEER research database.

## Methods

### Database and cohort definition

**Fig. 1:**
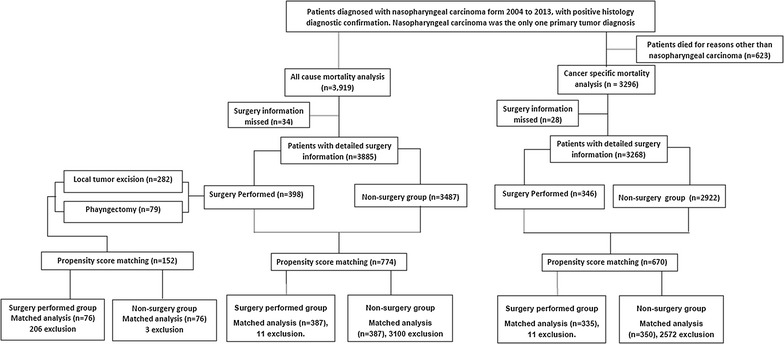
Flow diagram

The SEER*Stat database, which was released by the Surveillance Research Program at NCI in 2016, was used as the data source in the present study [17]. 10,193 patients diagnosed as nasopharyngeal carcinoma (The 3rd edition of International Classification of Diseases for Oncology (ICD-O-3)/WHO 2008 and Behavior code ICD-O-3: malignant) were identified in the SEER 18 Research Data + Hurricane Katrina Impacted Louisiana Cases, Nov 2015 Sub (1973–2013 varying) incidence database. SEER Registry collects stage at diagnosis, age at diagnosis, cancer type, gender, race and surgery/radiation treatment information. Because the database include information of detailed stage (2004 AJCC 6th and 2010 AJCC 7th) information from 2004, so we only included histologically confirmed cases (3919) diagnosed from 2004 to 2013 (Additional file 1: Table S1). Based on information regarding surgery, the patients were categorized into groups: surgery performed group, surgery recommended but not performed group, and surgery not recommended group. Non-surgery group was the combination of surgery recommended but not performed group and surgery not recommended group (Fig. 1). Based on radiation therapy information, patients were also divided into radiation group and non-radiation group.

### Histology categories

SEER data were collected and reported using data items and codes as documented by the North American Association of Central Cancer Registry (NAACCR) [18]. Histology was coded according to ICD-O-3. Histology categories according to ICD-0-3 included in this study were codes 8020/3 (carcinoma undifferentiated) and 8021/3 (carcinoma anaplastic) for undifferentiated, 8072/3 (squamous cell carcinoma, large cell, nonkeratinizing) and 8073/3 (squamous cell carcinoma, small cell, nonkeratinizing) for non-keratinizing squamous, 8071/3 (squamous cell carcinoma, keratinizing) for keratinizing squamous cancer histology. These three histology categories were evaluated for the risk of mortality. Other included histology types in study were 8074/3 (squamous cell carcinoma, spindle cell), 8075/3 (squamous cell carcinoma, adenoid) and 8010/3 (carcinoma, NOS) (Additional file 1: Table S1).

### Statistical analysis

Patients were followed up until December 2013. The primary outcomes measure all-cause mortality and nasopharyngeal carcinoma-specific mortality. The candidate risk factors included surgery, radiation, age, sex, race, differentiation grade, histology, TMN stage and AJCC stage. Numeric variables were summarized as the mean (standard deviation) and median (interquartile range). Categorical variables were reported as counts (percentage). An analysis of variance was used to compare continuous variables with symmetric distributions across the surgery subgroups and radiation subgroups. Chi square tests or Fisher’s exact tests (n < 5) were used to compare categorical variables between the treatment subgroups. The Kaplan–Meier method was used to plot the survival distributions, and the log-rank test was used to assess differences in survival experience among the subgroups. The Cox proportional hazards regression was performed to estimate the hazard ratio to identify the risk factors for nasopharyngeal carcinoma-specific mortality and all-cause mortality. To further adjust for potential baseline confounders, a propensity score matching was carried out. To evaluate the different effect of surgery or radiation for different clinical subgroups by the stratification variables, stratified Cox regression models were performed. A receiver operating characteristic (ROC) curve was also calculated to determine the optimal age cutoff that maximizes sensitivity and specificity in predicting mortality. All tests of hypotheses were two-tailed and conducted at a significance level of 0.05. Statistical analyses were conducted using SAS 9.4.

## Results

### Demographic and clinical characteristics of the nasopharyngeal carcinoma patients in 2004–2013

According to US Census Bureau (http://www.census.gov/), 72.4% US population were White Americans, 12.6% were African-American (AA), and only 4.8% were Asian. SEER database collected data from 30% US population. In this study, among 3919 histologically confirmed cases diagnosed in US from 2004 to 2013, 1784 (48.58%) were White Americans, 448 (12.20%) were Black Americans, and 1440 (39.22%) patients were Chinese or other Asian Americans (Table 1). Asian Americans had a much higher incidence to have NPC than White Americans.

**Table 1:** Characteristics for nasopharyngeal carcinoma patients stratified by surgery and radiation treatment

Covariate	Level	Overall (n = 3919)	Surgery			Radiation		
			Performed (n = 398)	None or refused (n = 3480)	p value	Radiation (n = 3114)	None or Refused (n = 657)	p value
Age		53.64 ± 15.26 54 (44, 63)	52.03 ± 16.43 53 (42, 63)	53.79 ± 15.08 54 (45, 63)	0.04	52.54 ± 14.87 53 (44,62)	58.40 ± 15.95 58 (49, 69)	<0.0001
Survival months		36.97 ± 32.47 27 (9, 59)	43.34 ± 32.56 38.5 (15, 64)	36.24 ± 32.37 26 (9, 58)	<0.0001	40.17 ± 32.49 31 (12, 62)	22.67 ± 28.58 10 (2, 32)	<0.0001
All-cause mortality	No	2471 (63.05%)	284 (71.36%)	2169 (62.33%)	0.0004	2125 (68.24%)	267 (40.64%)	<0.0001
	Yes	1448 (36.95%)	114 (28.64%)	1311 (37.67%)		989 (31.76%)	390 (59.36%)	
NPC-specific death	No	2471 (74.97%)	284 (82.08%)	2169 (74.36%)	0.002	2125 (79.06%)	267 (54.83%)	<0.0001
	Yes	825 (25.03%)	62 (17.92%)	748 (25.64%)		563 (20.94%)	220 (45.17%)	
Sex	Male	2798 (71.40%)	271 (68.09%)	2502 (71.90%)	0.11	2214 (71.10%)	473 (71.99%)	0.64
	Female	1121 (28.60%)	127 (31.91%)	978 (28.10%)		900 (28.90%)	184 (28.01%)	
Race	White	1784 (48.58%)	233 (61.64%)	1528 (46.96%)	<0.0001	1387 (47.35%)	332 (54.52%)	0.0009
	Black	448 (12.20%)	50 (13.23%)	393 (12.08%)		351 (11.98%)	82 (13.46%)	
	Chinese	669 (18.22%)	32 (8.47%)	631 (19.39%)		559 (19.09%)	85 (13.96%)	
	Other Asian	771 (21.00%)	63 (16.67%)	702 (21.67%)		632 (21.58%)	110 (18.06%)	
Grade	Well-differentiated	61 (2.23%)	13 (4.15%)	46 (1.91%)	0.04	46 (2.04%)	13 (3.19%)	<0.0001
	Moderate	359 (13.11%)	48 (15.34%)	307 (12.77%)		261 (11.58%)	78 (19.12%)	
	Poorly differentiated	1256 (45.87%)	135 (43.13%)	1113 (46.30%)		1037 (46.01%)	190 (46.57%)	
	Undifferentiated	1062 (38.79%)	117 (37.38%)	938 (39.02%)		910 (40.37%)	127 (31.13%)	
Histology	Keratinizing	184 (16.93%)	21 (17.21%)	162 (16.88%)	0.93	139 (15.48%)	41 (28.28%)	0.0002
	Non-keratinizing	903 (83.07%)	101 (82.79%)	798 (83.13%)		759 (84.52%)	104 (71.72%)	
T-stage	T0	31 (0.89%)	4 (1.10%)	27 (0.87%)	0.003	25 (0.86%)	4 (0.88%)	0.01
	T1	1126 (32.38%)	150 (41.10%)	971 (31.42%)		945 (32.44%)	140 (30.70%)	
	T2	786 (22.61%)	81 (22.19%)	699 (22.62%)		692 (23.76%)	80 (17.54%)	
	T3	729 (20.97%)	61 (16.71%)	662 (21.42%)		597 (20.49%)	110 (24.12%)	
	T4	805 (23.15%)	69 (18.90%)	731 (23.66%)		654 (22.45%)	122 (26.75%)	
N-stage	N0	856 (23.72%)	141 (38.42%)	708 (21.97%)	<0.0001	663 (22.19%)	166 (32.74%)	<0.0001
	N1	1270 (35.19%)	127 (34.60%)	1137 (35.29%)		1052 (35.21%)	177 (34.91%)	
	N2	997 (27.63%)	72 (19.62%)	920 (28.55%)		874 (29.25%)	93 (18.34%)	
	N3	286 (13.47%)	27 (7.36%)	457 (14.18%)		399 (13.35%)	71 (14.00%)	
M-stage	M0	3261 (88.98%)	346 (95.05%)	2889 (88.32%)	<0.0001	2748 (91.63%)	408 (74.59%)	<0.0001
	M1	404 (11.02%)	18 (4.95%)	382 (11.68%)		251 (8.37%)	139 (25.41%)	
AJCC stage	I	292 (8.51%)	65 (18.52%)	224 (7.32%)	<0.0001	229 (7.98%)	57 (12.39%)	<0.0001
	II	741 (21.60%)	92 (26.21%)	647 (21.14%)		662 (23.06%)	58 (12.61%)	
	III	969 (28.24%)	85 (24.22%)	879 (28.73%)		859 (29.92%)	87 (18.93%)	
	IV	1429 (41.65%)	109 (31.05%)	1310 (42.81%)		1121 (39.05%)	258 (56.09%)	
Radiation	No	657 (17.42%)	62 (16.02%)	591 (17.57%)	0.45			
	Yes	3114 (82.58%)	325 (83.98%)	2772 (82.43%)				
Surgery	None or refused	3480 (89.74%)				2772 (89.51%)	591 (90.51%)	0.45
	Performed	398 (10.26%)				325 (10.49%)	62 (9.49%)	

Of the 3919 cases included in the analysis (Fig. 1), 398 cases underwent surgery as defined above (surgery group), whereas 3487 patients refused or were not recommended for surgery (non-surgery group). 3114 cases underwent radiotherapy (radiation group), whereas 657 cases did not (non-radiation group). Treatment characteristics across groups were outlined in Table 1, showing a significant younger age, more female, white, well/moderate differentiated, T1, N0, M0, and AJCC I/II stage patients in surgery group. Also, more patients with younger age, Asian, undifferentiated, non-keratinizing tumors, N2/M0, AJCC II/III stage were in radiation group. ROC curve determined the age of diagnosis at 60 year as the optional cutoff age that maximized sensitivity and specificity for predicting both nasopharyngeal carcinoma-specific mortality and all-cause mortality (Additional file 2: Figures S1 and S2). As for surgery type, among 398 cases who underwent surgery, 282 (70.85%) received local tumor excision and 79 (20.31%) received pharyngectomy (Table 1).

### Clinical outcomes

The overall and cancer-specific survival curves were shown in Additional file 3: Figure S3A and B. The 9-year estimated overall survival rates and cancer-specific survival rates were 49.29% and 65.81%, respectively (Additional file 3: Tables S4 and S5). The survival months were longer in surgery or radiation group, compared to non-surgery and non-radiation group, respectively (p < 0.0001, Table 1). Both all-cause mortality rates and nasopharyngeal carcinoma-specific mortality rates were significantly lower in surgery and radiation groups, compared to non-surgery and non-radiation groups, respectively (p < 0.0001, Table 1).

**Fig. 2:**
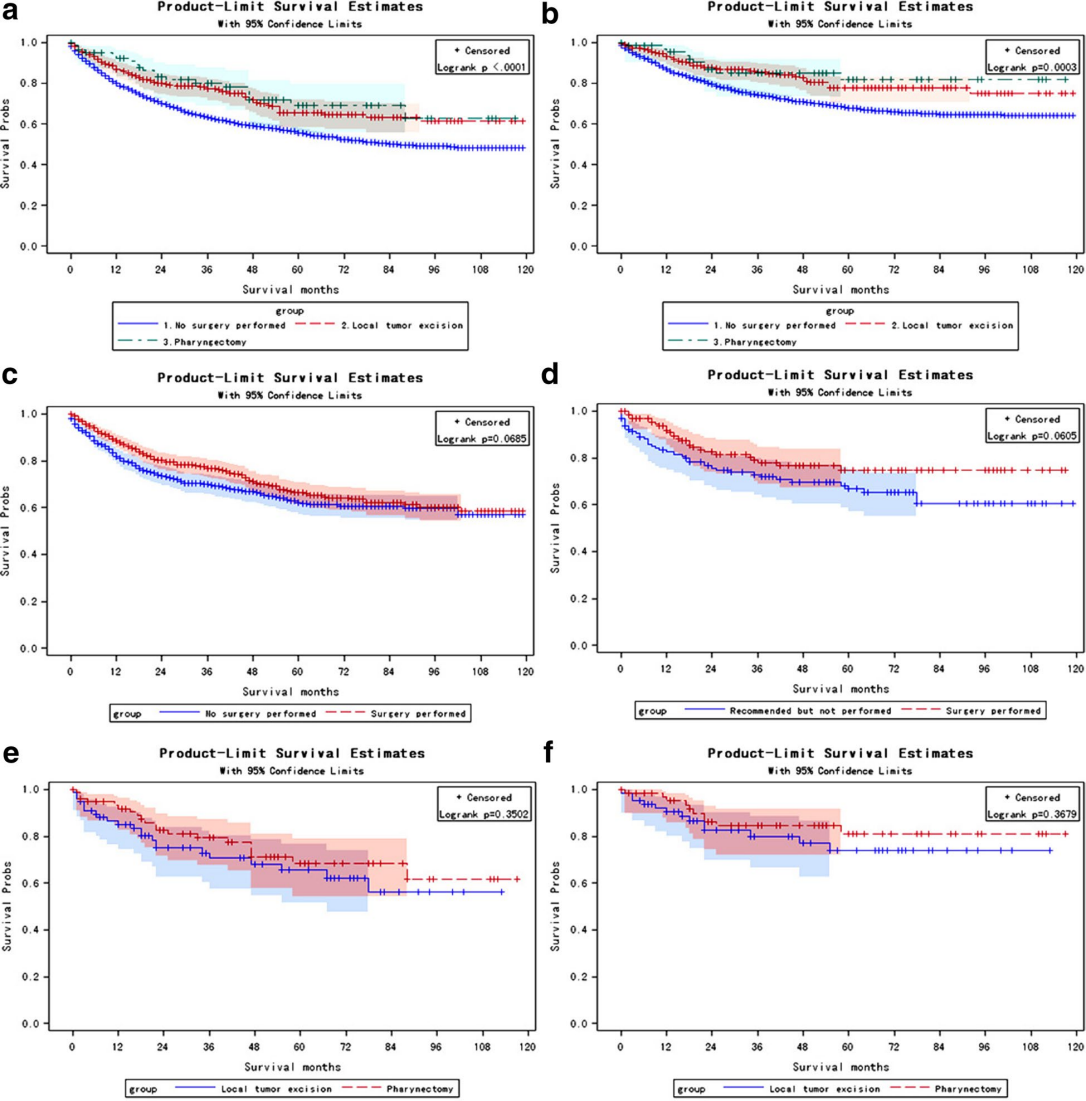
Kaplan–Meier curves stratified by surgery. Y-axis label “Survival probs” means survival probabilities. **a**, **b** Kaplan–Meier curves among unmatched patients stratified by surgery types for all-cause death (**a**, Log rank test p < 0.0001) and nasopharyngeal carcinoma-specific death (**b**, Log rank test p = 0.0003). **c**, **d** Kaplan–Meier curves among matched patients stratified by surgery/non-surgery for all-cause death (**c**, Log rank test p = 0.0685) and nasopharyngeal carcinoma-specific death (**d**, Log-rank test p = 0.0605). **e**, **f** Kaplan Meier curves among matched patients stratified by surgery type local tumor excision and pharyngectomy for all-cause death (**e**, Log rank test p = 0.3502) and nasopharyngeal carcinoma-specific death (**f**, Log-rank test p = 0.3679)

The prognostic impact of surgery on all cause mortality and cancer-specific mortality was outlined in Fig. 2. Kaplan–Meier curves showed a higher overall survival in patients undergoing local tumor excision or pharyngectomy compared to patients in non-surgery group (p < 0.0001, Fig. 2a). Similar result was detected for cancer-specific survival (p < 0.0003, Fig. 2c).

### Risk factors for all-cause mortality and nasopharyngeal carcinoma-specific mortality

Univariate COX regression analysis showed a significant protective effect for local tumor excision (*HR = 0.646, 95% CI 0.514–0.812; **HR = 0.593, 95% CI 0.434–0.809) and pharyngectomy (*HR = 0.522, 95% CI 0.355–0.859; **HR = 0.480, 95% CI 0.257–0.895) and against all-cause death (*p = 0.0002 and 0.008, respectively) and nasopharyngeal carcinoma-specific death (**p = 0.001 and 0.02, respectively). But multivariate COX regression analysis showed no significant differences between the surgery group and non-surgery group in the risk of both all-cause death and nasopharyngeal carcinoma-specific death. Both univariate and multivariate COX analyses showed significant difference between radiotherapy group and non-radiation group in the risk of both all-cause death and nasopharyngeal carcinoma-specific death (p < 0.0001). The other parameters investigated, including age, sex (male), Asian, un-differentiation, histology type, T stage, N/M stage, AJCC stage, were influential factors for both all-cause mortality and cancer-specific mortality in univariate COX model (Table 2).

**Table 2:** Risk factors for survival: outcome is all-cause mortality and nasopharyngeal carcinoma specific mortality

Variables	Level^a^	All-cause mortality				Cancer-specific mortality			
		Univariate Cox regression		Multivariate Cox regression		Univariate Cox regression		Multivariate Cox regression	
		Hazard ratio (95% CI)	p value	Hazard ratio (95% CI)	p value	Hazard ratio (95% CI)	p value	Hazard ratio (95% CI)	p value
Age		1.040 (1.036, 1.043)	<0.0001	1.036 (1.027, 1.045)	<0.0001	1.041 (1.036, 1.046)	<0.0001	1.033 (1.021, 1.045)	<0.0001
Sex	Male	1.184 (1.063, 1.318)	0.002	1.325 (1.000, 1.756)	0.05	1.198 (1.037, 1.383)	0.01	1.240 (0.846, 1.816)	0.27
	Female	Ref		Res		Ref		Ref	
Race	White	Ref		Ref		Ref		Ref	
	Black	0.991 (0.858, 1.144)	0.90	1.353 (0.938, 1.945)	0.11	0.897 (0.724, 1.111)	0.32	1.287 (0.756, 2.191)	0.35
	Chinese	0.494 (0.421, 0.578)	<0.0001	0.612 (0.430, 0.872)	0.007	0.693 (0.574, 0.836)	0.0001	0.782 (0.503, 1.216)	0.24
	Other Asian	0.615 (0.535, 0.707)	<0.0001	0.593 (0.421, 0.836)	0.003	0.759 (0.635, 0.907)	0.002	0.675 (0.435, 1.048)	0.08
Grade	Well	Ref		Ref		Ref		Ref	
	Moderately	1.292 (0.910, 1.834)	0.15	1.191 (0.600, 2.364)	0.62	1.124 (0.685, 1.844)	0.64	1.234 (0.354, 4.303)	0.72
	Poorly	0.768 (0.550, 1.072)	0.12	0.879 (0.451, 1.713)	0.71	0.834 (0.525, 1.326)	0.44	0.999 (0.305, 3.268)	1.00
	Undifferentiated	0.504 (0.359, 0.707)	<0.0001	0.829 (0.399, 1.722)	0.61	0.535 (0.334, 0.855)	0.009	0.866 (0.253, 2.959)	0.82
Histology	Keratinizing	Ref		Ref		Ref		Ref	
	Non-keratinizing	0.462 (0.337, 0.538)	<0.0001	0.513 (0.355, 0.740)	0.0004	0.530 (0.382, 0.735)	0.0001	0.721 (0.415, 1.254)	0.25
	Non-differentiated	0.338 (0.259, 0.440)	<0.0001	0.531 (0.316, 0.892)	0.02	0.328 (0.224, 0.479)	<0.0001	0.575 (0.276, 1.196)	0.14
T-stage	T0	1.125 (0.617, 2.051)	0.70	4.438 (0.555, 35.496)	0.16	0.787 (0.292, 2.121)	0.64	–	
	T1	Ref		Ref		Ref		Ref	
	T2	1.298 (1.108, 1.519)	0.001	1.069 (0.727, 1.572)	0.54	1.455 (1.174, 1.803)	0.0006	1.140 (0.673, 1.931)	0.63
	T3	2.024 (1.742, 2.351)	<0.0001	1.312 (0.858, 2.007)	0.21	2.327 (1.898, 2.853)	<0.0001	1.520 (0.866, 2.666)	0.14
	T4	2.438 (2.109, 2.818)	<0.0001	1.822 (1.125, 2.951)	0.01	2.877 (2.364, 3.501)	<0.0001	1.943 (1.035, 3.648)	0.04
N-stage	N0	Ref		Ref		Ref		Ref	
	N1	0.835 (0.729, 0.956)	0.009	0.973 (0.683, 1.387)	0.88	0.867 (0.720, 1.044)	0.13	0.964 (0.594, 1.563)	0.88
	N2	0.893 (0.775, 1.030)	0.12	0.926 (0.626, 1.371)	0.70	0.989 (0.816, 1.198)	0.90	1.071 (0.645, 1.778)	0.79
	N3	1.440 (1.226, 1.692)	<0.0001	1.784 (1.095, 2.907)	0.02	1.714 (1.385, 2.120)	<0.0001	1.666 (0.879, 3.158)	0.12
M-stage	M0	Ref		Ref		Ref		Ref	
	M1	3.469 (3.065, 33.926)	<0.0001	1.862 (1.224, 2.831)	0.004	4.487 (3.830, 5.255)	<0.0001	1.908 (1.131, 3.218)	0.02
AJCC stage	I	Ref		Ref		Ref		Ref	
	II	1.394 (1.051, 1.848)	0.02	1.102 (0.551, 2/202)	0.78	1.805 (1.157, 2.817)	0.009	1.168 (0.419, 3.258)	0.77
	III	1.915 (1.463, 2.505)	<0.0001	1.820 (0.901, 3.678)	0.10	2.885 (1.888, 4.409)	<0.0001	1.858 (0.673, 5.126)	0.23
	IV	3.994 (3.090, 5.164)	<0.0001	1.968 (0.938, 4.131)	0.07	6.571 (4.359, 9.904)	<0.0001	2.606 (0.905,7.499)	0.08
Radiation	None or refused	Ref		Ref		Ref		Ref	
	Yes	0.333 (0.300, 0.371)	<0.0001	0.364 (0.264, 0.502)	<0.0001	0.319 (0.276, 0.368)	<0.0001	0.380 (0.244, 0.593)	<0.0001
Surgery	None	Ref		Ref		Ref		Ref	
	Local tumor destruction	0.794 (0.112, 5.644)	0.82	0.871 (0.544, 1.393)	0.56	1.215 (0.171, 8.635)	0.85	0.545 (0.065, 4.546)	0.58
	Local tumor excision^a^	0.646 (0.514, 0.812)	0.0002	1.019 (0.442, 2.352)	0.96	0.593 (0.434, 0.809)	0.001	0.829 (0.416, 1.653)	0.59
	Pharyngectomy^b^	0.522 (0.355, 0.859)	0.008	0.457 (0.063, 3.312)	0.44	0.480 (0.257, 0.895)	0.02	1.126 (0.399, 3.173)	0.82

^a^The risk of all-cause mortality among non-surgery group (surgery recommended but not performed group + surgery not recommended group), compared with surgery group

^b^The risk of nasopharyngeal carcinoma-specific mortality in non-surgery group, compared with surgery group

### Adjusting for patient characteristics using propensity score matching

To reduce the confounding bias of patients’ selection for surgery and non-surgery group, we performed propensity score matching. Propensity score matching was carried out regarding age, sex, race, differentiation grades, T/N/M stage, AJCC stage, histology type and radiation therapy. The standardized differences for matched variables decreased to less than 0.1 and propensity score improved to near equality after matching (Additional file 4: Figure S4).

As shown in Fig. 1, in overall dataset, propensity score matching procedure resulted in the exclusion of 3145 patients (11 patients in the surgery group and, 3100 in non-surgery group, and 34 with missed surgery information) who lacked a propensity score match. In dataset excluding deaths for other causes, propensity score matching procedure resulted in the exclusion of 2583 patients (11 patients in the surgery group and, 2572 in non-surgery group, and 28 with missed surgery information) who lacked a propensity score match. In the Cox regression model after propensity score matching, surgery remained a marginal prognostic factor for both overall mortality (HR = 0.816, 95% CI 0.656–1.015, p = 0.07) and cancer-specific mortality (HR = 0.749, 95% CI 0.552–1.018, p = 0.06). Kaplan–Meier analysis showed a marginally significant difference between surgery and non-surgery groups for overall mortality (Log-rank p = 0.0685, Fig. 2d) and cancer-specific mortality (Log-rank p = 0.0638, Fig. 2b).

### Stratified Cox model

**Fig. 3:**
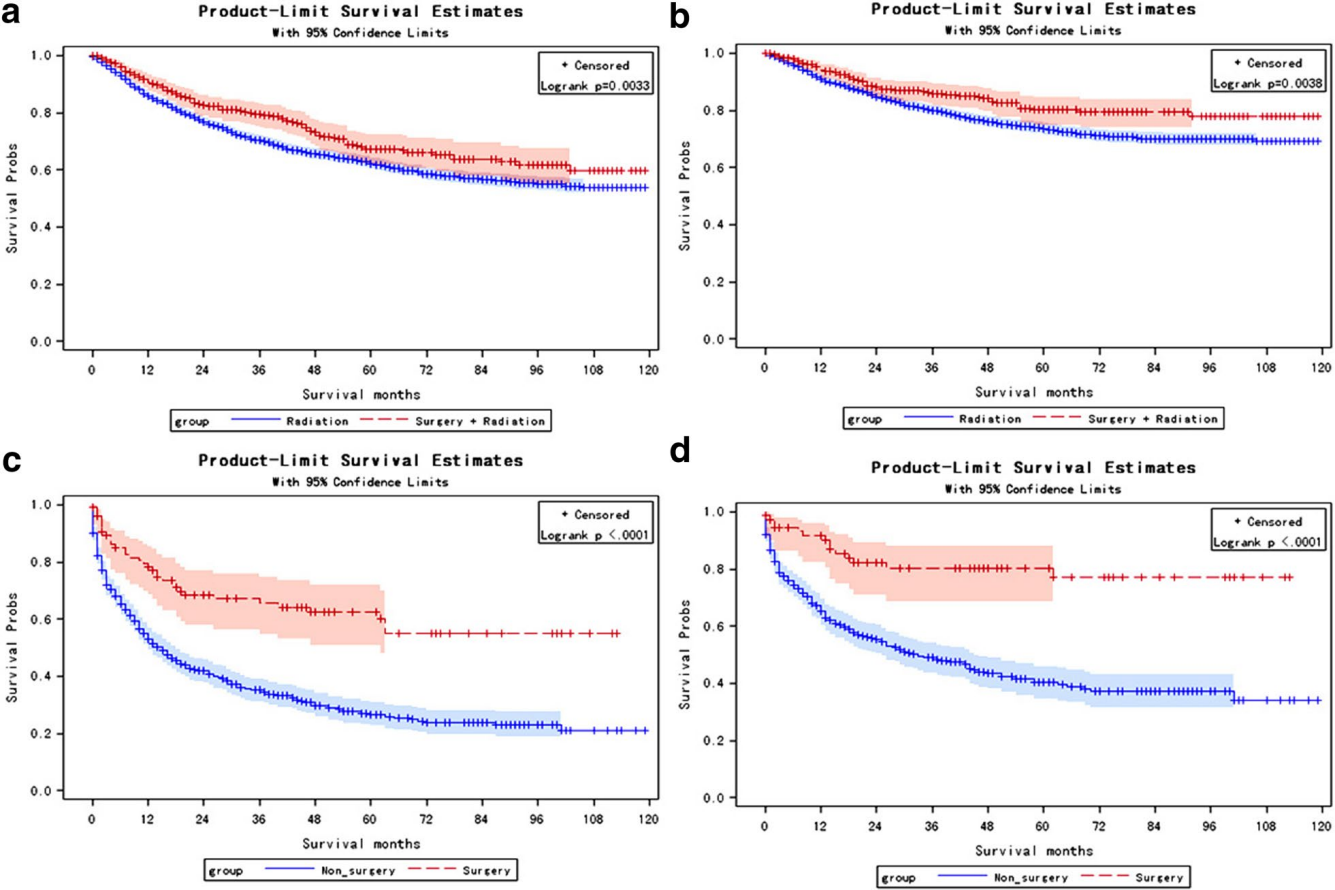
Kaplan Meier curves among patients stratified by surgery for nasopharyngeal carcinoma-specific death and all-cause death in patients with RT (**a** all-cause death, Log-rank test p = 0.0033; **b** cancer-specific death Log rank test p = 0.0038) and in patients without RT (**c** all-cause death, Log-rank test p < 0.0001; **d** cancer-specific death Log rank test p < 0.0001). Y-axis label “Survival probs” means survival probabilities

To see whether the effect of surgery or radiation was different for subgroups by the stratification variables, stratified Cox regression models were used. As demonstrated in Table 3, compared to nonsurgery group, patients with surgery was strongly associated with a better nasopharyngeal carcinoma-specific survival in subgroups of patients with age >60 year, white, AJCC stage III, T3, N1, M0, keratinizing/non-keratinizing tumor with moderately differentiated. As for surgery type, local tumor excision performed better in subgroup of patients with T3, M1, undifferentiated tumor (Table 3). In propensity score matching analysis, local tumor excision had no significant priority than pharyngectomy (Fig. 2e, f). In both RT and non-RT group, patients with surgery performed had a better prognostic than non-surgery group (Fig. 3). These findings suggested that surgery should be recommended in white patients with moderately differentiated tumor in T3, N1, M0, AJCC III stage, no matter about the age and gender.

**Table 3:** Stratified Cox regression analysis for risk subgroup factors of nasopharyngeal carcinoma-specific death related to surgery treatment

Variable	Levels	Local tumor excision vs non-surgery		Pharyngectomy vs non-surgery		Surgery vs nonsurgery		p*
		HR (95% CI)	p value	HR (95% CI)	p value	HR (95% CI)	p value	
Age (year)	≥60	0.71 [0.48, 1.05]	0.09	0.72 [0.36, 1.45]	0.36	0.79 [0.58, 1.09]	0.15	0.25
	<60	0.44 [0.27, 0.73]	0.002	0.20 [0.05, 0.79]	0.02	0.42 [0.27, 0.65]	0.0001	
Sex	Male	0.42 [0.23, 0.76]	0.004	0.92 [0.34, 2.46]	0.87	0.44 [0.26, 0.74]	0.002	0.25
	Female	0.67 [0.47, 0.96]	0.03	0.36 [0.16, 0.79]	0.01	0.70 [0.52, 0.94]	0.02	
Race	White (n = 91)	0.46 [0.29, 0.71]	0.0002	0.40 [0.18, 0.89]	0.03	0.51 [0.36, 0.72]	0.0002	<0.01
	Black (n = 656)	0.35 [0.11, 1.09]	0.07	0.92 [0.23, 3.73]	0.90	0.54 [0.25, 1.18]	0.12	
	Asian (n = 1806)	0.97 [0.58, 1.63]	0.91	0.24 [0.03, 2.02]	0.16	0.77 [0.47, 1.29]	0.32	
AJCC stage	I	1.14 [0.41,3.14]	0.80	1.14 [0.15, 8.62]	0.90	1.14 [0.44, 2.93]	0.79	0.42
	II	0.89 [0.43, 1.85]	0.76	0.86 [0.21, 3.51]	0.84	0.86 [0.46, 1.62]	0.64	
	III	0.35 [0.13, 0.93]	0.04	0.26 [0.04, 1.82]	0.17	0.37 [0.17, 0.84]	0.02	
	IV	0.85 [0.54, 1.35]	0.49	0.57 [0.25, 1.28]	0.17	0.76 [0.52, 1.11]	0.16	
T stage	T1	0.69 [0.38, 1.25]	0.22	0.55 [0.17, 1.72]	0.30	0.62 [0.37, 1.07]	0.08	0.06
	T2	0.58 [0.27, 1.23]	0.16	0.29 [0.04, 2.10]	0.22	0.58 [0.31, 1.10]	0.10	
	T3	0.16 [0.04, 0.63]	0.009	0.68 [0.17, 2.76]	0.59	0.29 [0.12, 0.71]	0.007	
	T4	1.09 [0.64, 1.88]	0.75	0.80 [0.30, 2.16]	0.66	1.11 [0.71, 1.72]	0.65	
N stage	N0	0.63 [0.38, 1.04]	0.07	0.59 [0.22, 1.60]	0.30	0.71 [0.46, 1.08]	0.11	0.86
	N1	0.55 [0.29, 1.03]	0.06	0.57 [0.18, 1.79]	0.34	0.52 [0.30, 0.89]	0.02	
	N2	0.55 [0.24, 1.23]	0.15	0.61 [0.20, 1.91]	0.40	0.61 [0.33, 1.16]	0.13	
	N3	0.67 [0.21, 2.12]	0.50	–	–	0.42 [0.15, 1.13]	0.09	
M stage	M0	0.71 [0.50, 1.00]	0.05	0.53 [0.26, 1.06]	0.07	0.69 [0.51, 0.93]	0.01	0.84
	M1	0.24 [0.06, 0.97]	0.05	1.63 [0.40, 6.61]	0.49	0.45 [0.18, 1.09]	0.08	
Grade	Well	0.46 [0.10, 2.02]	0.30	1.13 [0.14, 8.87]	0.91	0.57 [0.16, 1.99]	0.38	0.07
	Moderately	0.60 [0.24, 1.48]	0.27	–	–	0.36 [0.16, 0.82]	0.02	
	Poorly	0.95 [0.62, 1.47]	0.82	0.46 [0.15, 1.44]	0.18	0.85 [0.57, 1.25]	0.40	
	Undifferentiated	0.40 [0.19, 0.85]	0.02	1.78 [0.79, 4.04]	0.16	0.65 [0.37, 1.12	0.12	
Histology	keratinizing	0.74 [0.51, 1.06]	0.09	0.31 [0.13, 0.76]	0.01	0.68 [0.50, 0.93]	0.01	0.02
	Nonkeratinizing	0.39 [0.18, 0.83]	0.01	0.61 [0.20, 1.92]	0.40	0.45 [0.24, 0.82]	0.01	
	Undifferentiated	0.54 [0.20, 1.48]	0.23	2.36 [0.58, 9.66]	0.23	0.81 [0.37, 1.78]	0.60	
Radiation	No	0.34 [0.16, 0.72]	0.005	0.16 [0.02, 1.12]	0.06	0.35 [0.19, 0.64]	0.0007	0.03
	Yes	0.69 [0.48, 0.98]	0.04	0.64 [0.33, 1.23]	0.18	0.71 [0.53, 0.96]	0.02	

p* indicated the comparison of surgery vs non-surgery among subgroups

### Treatment trend of NPC

**Fig. 4:**
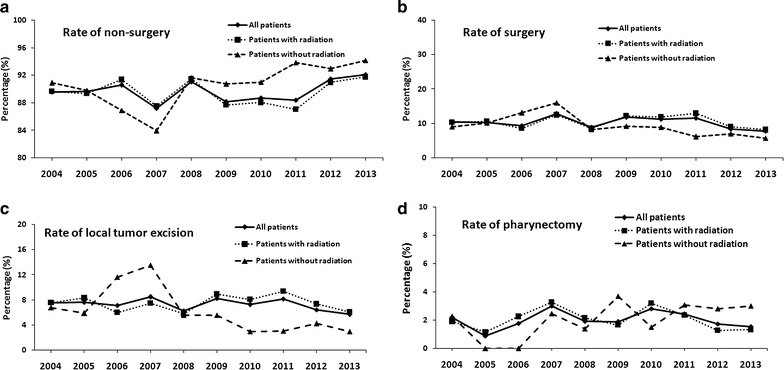
Treatment trend of NPC. Trend of nonsurgery (**a**) and surgery (**b**) in all patients (*line*), patients with radiation (*dot line*) and patients without radiation (*dash line*). **c** Trend of local tumor excision in all patients (*line*), patients with radiation (*dot line*) and patients without radiation (*dash line*). **d** Trend of pharynectomy in all patients (*line*), patients with radiation (*dot line*) and patients without radiation (*dash line*)

Even patients with surgery showed a better overall survival and cancer-specific survival in NPC patients, the overall portion of surgery patients decreased from 10.41% in 2004 to 7.86% in 2013 (Fig. 4b). The rate of surgery in patients without radiation fluctuated from 9.09% in 2004 to 16.05% in 2007 and then decreased to 5.8% in 2013. The portion of nonsurgery patients decreased from 89.59% in 2004 to 87.22% 2007, and then increased to 92.14% in 2013 (Fig. 4a). As for two surgery types in patients without radiation, the rate of local tumor excision increased from 6.82% in 2004 to 13.58% in 2007, and then decreased to 2.99% in 2013 (Fig. 4c), while the rate of pharyngectomy decreased from 2.27% in 2004 to 0% in 2007, and then recovered to 2.99% in 2013 (Fig. 4d). The portion of these two types of surgery among all patients or patients with radiation decreased slightly about 20–30% over 2004–2013 (Fig. 4c, d).

## Discussion

Over the last three decades, the incidence rates of nasopharyngeal carcinoma decreased significantly in southern and eastern Asian [19], while the incidence rate in the United States have remained almost unchanged [20]. The survival rates for each AJCC stage and histological subtype have exhibited considerable improvement over time, especially in Asians due to more favorable undifferentiated histology in this group [21]. The present study is the first population-based analysis using propensity score matching methods to provide evidence of a positive impact of primary tumor surgery on mortality in nasopharyngeal carcinoma patients.

Nasopharyngeal carcinoma has a high propensity for local recurrences after radiation for locally advanced disease. Primary tumor surgery was recommended for local and regional residual or recurrent tumors after radiotherapy [22], without skull base and brain nerves damage and distant metastasis [23]. Interestingly, in this study, among patients who underwent surgery, 325 (83.98%) had received radiation beam, and 310 (95.38%) of them received surgery before radiation. 223 (79.08%) of 282 local tumor excision patients and 61 (77.22%) of 79 pharyngectomy patients underwent surgery before radiation. Stratified Cox regression analysis demonstrated that in radiation group, patients with surgery had lower risk to cancer-specific death than non-surgery patients (HR = 0.71, 95% CI 0.53–0.96, p = 0.02, Table 3). Due to small sample size, patients with pharyngectomy did not show a significant better prognostic than non-surgery group in both radiation and non-radiation groups, but the estimated HRs were much lower than 1 (Table 3). Survival analysis also showed that patients with surgery had a significant better prognostic than non-surgery group in both RT (Fig. 3a, b) and non-RT groups (Fig. 3c, d). Therefore, we suggested that most primary NPC surgery performed before radiation and benefited the survival.

According to NCCN guidelines, AJCC stage I (T1N0M0) patients may be treated with definitive RT alone. For stage II–IVB (T1, N1–3 and T2–4, and N lesions) patients, concurrent chemotherapy plus RT with/without adjuvant chemotherapy are recommended [11]. Stage IVC (any T, any N, M1) patients, the treatment options include clinical trial, platinum-based chemotherapy, and concurrent chemo/RT [24, 25]. The 5-year disease-specific survival is 100% for stage I, 95% for II, 90% for III, 67% for IVA, 68% for IVB and 18% for IVC [26]. No primary tumor surgery is recommended in NCCN guidelines. However, according to SEER database, there is a deviation from NCCN guidelines, because 398 cases received surgery from 2004 to 2013. Three patients received local tumor destruction. 282 patients received local tumor excision. 79 patients received pharyngectomy (including pharyngectomy alone, pharyngectomy with laryngectomy or removal of contiguous bone tissue, and radical pharyngectomy). 34 surgery patients had no information about surgery type. As shown in Additional file 5: Figure S5 and Tables S6, S7, NPC-specific survival in surgery group was significantly higher than non-surgery group (p = 0.0002). The 5-year disease-specific survival is 77.28% for patients with surgery, compared to 67.83% for non-surgery patients (Additional file 5: Table S7).

In the present study, AJCC 6th edition was applied for the patients diagnosed between 2004 and 2009, and 7th edition was applied from 2010 to 2013. In the present study, we evaluated the treatment strategies based on clinical characteristics (AJCC TNM stages, histology, and differentiation grade) at diagnosis. Stratified Cox regression showed that AJCC stage III (T1–T3N2, T2–3N0, T3 N1) patients in surgery group had a better outcome than non-surgery group for nasopharyngeal carcinoma-specific survival (Table 3). In further stratified Cox model (Table 3), surgery group showed a significantly lower risk of nasopharyngeal carcinoma-specific death (HR = 0.37, 95% CI 0.17–0.84, p = 0.02) in AJCC stage III patients.

Without surgery, the 5-year NPC-specific survival rate was 67.83%. Still 32.17% patients died because of NPC treatment failure (Additional file 5: Table S7). Even radiation could eliminate the localized tumors, some patients still died due to tumor recurrence. For recurrent tumors, advances in skull base surgery make possible the effective control of primary recurrence of nasopharyngeal carcinoma for patients with local and regional recurrent T1 (rT1) and rT2 stages [27]. According to AJCC staging, T1/T2 tumor is confined to the nasopharynx, oropharynx, nasal cavity and parapharynx. T3 tumor involves bony structures of skull base, and T4 tumor has intracranial extension and/or the involvement of cranial nerves. As shown in Table 3, T3 patients were recommended for surgery (HR = 0.29, 95% CI 0.12–0.71, p = 0.007), especially for local tumor excision (HR = 0.16, 95% CI 0.04–0.63, p = 0.009), but not recommended for pharyngectomy (HR = 0.68, 95% CI 0.17–2.76, p = 0.59). Besides T stages, N1 and M0 subgroups were also significantly benefited from surgery treatment, compared to non-surgery group (HR = 0.52, 95% CI 0.30–0.89, p = 0.02; HR = 0.69, 95% CI 0.51–0.93, p = 0.01). Local tumor excision (p < 0.1) had a better performance than pharyngectomy (p > 0.1) for N1 and M0 patients (Table 3). But, in analyses for propensity score matched data, local tumor excision did not show a significantly better outcome than pharyngectomy (Fig. 2e, f). Based on these findings, we hypothesized that surgery of primary NPC tumors might reduce tumor recurrence and thus benefit cancer-specific survival.

Nasopharyngeal carcinoma has complex histology origins. Based on a retrospective observational study from Sun Yat-Sen Cancer Center (Guangzhou, China), the 5-year OS rate of epithelial carcinoma, mixed sarcomatoid-epithelial carcinoma, sarcomatoid carcinoma, and squamous cell carcinoma were 79.4, 70.5, 59.6, and 42.6%, respectively [28]. Except for histology (ICD-O-3 code) records, SEER database also recorded the tumor differentiation grade, a much simpler system. Compared to differentiated squamous and non-keratinizing carcinoma, undifferentiated cancer has a significant better survival prognostics due to its high sensitivity to RT and chemotherapy [29]. Table 3 showed that in moderately-differentiated subgroup, surgery patients had significantly lower risk of cancer-specific death than non-surgery group (HR = 0.36, 95% CI 0.16–0.82, p = 0.02). In both keratinizing and non-keratinizing squamous tumor, surgery group also showed a better survival than surgery not recommended group (p = 0.01). In undifferentiated tumors, surgery had no such effect.

Racial disparity existed in nasopharyngeal carcinoma. Chinese patients have a higher survival rate due to their higher response to RT/chemotherapy [30]. But, a matched analysis showed that the biological behavior of NPC is relatively independent of race [31]. In this study, 61.64% white were in surgery group, which was significantly higher than the proportion of Chinese patients (18.22%, Table 1). Also, surgery showed significant benefit to white patients for cancer-specific survival (HR = 0.51, 95% CI 0.36–0.72, p = 0.0002, Table 3). Both local tumor excision and pharyngectomy had significant benefits to white patients (p = 0.0002 and 0.03, respectively, Table 3).

As demonstrated in propensity score analysis, there is no significant difference on survival between local tumor excision and pharyngectomy. So, it depends on real clinical situation to determine which type of surgery should be performed. For example, in T4 patients, both local tumor excision and pharyngectomy showed no benefits on survival. But in T3 patients, both local tumor excision and pharyngectomy showed significant benefits on survival. Stage III included T_1–2_N_2_M_0_ and T_3_N_0–2_M_0_. N2 patients were marginally sensitive to local tumor excision. But still some N2 patients belong to stage IV (any T, any N, M1). So, we further divided AJCC TNM stages into more detailed T stages, N stages and M stages. M. As shown in Table 3, local tumor excision significantly benefited T3 patients (HR = 0.16, 95% CI 0.04–0.63), and marginally benefited N2 patients (HR = 0.55, 95% CI 0.29–1.03). However, we should notice that the sample size of surgery patients in each subgroups was small (Table 1), especially for patients with pharyngectomy (only 79 patients between 2004 and 2013). To determine which type of surgery to be better, more samples with longer time accumulation are need in future study.

Retrospective observational study is prior than prospective randomized trials because it has no selection bias by entering good performance and small tumor patients possibly benefitting most from primary surgery in such trials [32]. This retrospective study used SEER registry data. SEER data have high completeness and accuracy, and are representing the entire patient population in the United States.

## Conclusion

The present study supports the favorable impact of surgery on clinical outcomes in patients with nasopharyngeal carcinoma. Most importantly, the benefit of tumor surgery is significant in subgroups of patients who are younger than 60 year, white, with T3, N1, M0, AJCC stage III, or moderately differentiated tumors. Survival analysis showed that patients with surgery had a better prognostics in both RT and non-RT patients.

## Additional files

**Additional file 1: Table S1.** Histology type stratified by differentiation grade.

**Additional file 2: Figure S1.** ROC for Age (AUC = 0.6423, P < 0.0001): outcome is all cause mortality (n = 4658). **Table S2.** 60 year is the optimal cutoff for age as a predictor of all-cause mortality. **Figure S2.** ROC for Age (AUC = 0.6423, P < 0.0001): outcome is cancer-specific mortality (n = 3894). **Table S3.** 60 year is the optimal cutoff for age as a predictor of all-cause mortality.

**Additional file 3: Figure S3.** Kaplan Meier Curve for all-cause mortality (A) and nasopharyngeal carcinoma-specific mortality (B). **Table S4.** Survival rate information for all cause mortality. **Table S5.** Survival rate information for nasopharyngeal carcinoma-specific mortality.

**Additional file 4: Figure S4.** Comparison of standardized differences (A, B) and propensity scores (C, D) in unmatched and matched samples. A, C: propensity score matching in overall dataset; B, D: propensity score matching of dataset excluding deaths with other reasons.

**Additional file 5: Figure S5.** Kaplan Meier Curve for all-cause mortality (A) and nasopharyngeal carcinoma-specific mortality (B) stratified by surgery. **Table S6.** Survival rate information for all cause mortality stratified by surgery. **Table S7.** Survival rate information for nasopharyngeal carcinoma-specific mortality stratified by surgery.

## Abbreviations

NPC: nasopharyngeal carcinoma
SEER: Epidemiology and End Results
HR: hazard ratio
NCI: National Cancer Institute
AJCC: American Joint Committee on Cancer
WHO: World Health Organization
K-NPSCC: keratinizing squamous cell carcinoma
NK-NPSCC: differentiated non-keratinizing squamous cell carcinoma
NCCN: National Comprehensive Cancer Network
RT: radiotherapy
NAACCR: North American Association of Central Cancer Registry
ICD-O-3: The 3rd edition of International Classification of Diseases for Oncology
ROC: receiver operating characteristic
