# Supplementary material for: Benefits of local tumor excision and pharyngectomy on the survival of nasopharyngeal carcinoma patients: a retrospective observational study based on SEER database
Source: J Transl Med. 2017 May 30;15:116. doi: 10.1186/s12967-017-1204-x (PMC5450381; doi:10.1186/s12967-017-1204-x)
Supplement: Supplementary file 1 — Additional file 1: Table S1. Histology type stratified by differentiation grade. [file 12967_2017_1204_MOESM1_ESM.docx]

**12967_2017_1204_MOESM1_ESM**

**Table S1: histology type stratified by differentiation grade.**

| Histological type | Grade (Differentiation) | | | | | Total |
| --- | --- | --- | --- | --- | --- | --- |
|  | Well | Moderately | Poorly | Undifferentiated | Unknown |  |
| 8000/3: Neoplasm, malignant | 0 | 0 | 2 | 3 | 79 | 84 (1.80%) |
| 8001/3: Tumor cells, malignant | 0 | 0 | 0 | 0 | 2 | 2 (0.04%) |
| 8010/3: Carcinoma, NOS | 4 | 6 | 276 | 245 | 436 | 967 (20.76%) |
| 8012/3: Large cell carcinoma, NOS | 0 | 0 | 6 | 40 | 3 | 13 (0.28%) |
| 8013/3: Large cell neuroendocrine carcinoma | 0 | 0 | 1 | 0 | 0 | 1 (0.02%) |
| 8020/3: Carcinoma, undifferentiated, NOS | 0 | 0 | 0 | 526 | 0 | 526 (11.29%) |
| 8021/3: Carcinoma, anaplastic, NOS | 0 | 0 | 0 | 3 | 0 | 3 (0.06%) |
| 8022/3: Pleomorphic carcinoma | 0 | 0 | 0 | 1 | 0 | 1 (0.02%) |
| 8030/3: Giant cell and spindle cell carcinoma | 0 | 0 | 1 | 0 | 0 | 1 (0.02%) |
| 8032/3: Spindle cell carcinoma, NOS | 1 | 0 | 2 | 3 | 3 | 9 (0.19%) |
| 8033/3: Pseudosarcomatous carcinoma | 0 | 0 | 1 | 0 | 0 | 1 (0.02%) |
| 8041/3: Small cell carcinoma, NOS | 0 | 0 | 5 | 0 | 11 | 16 (0.34%) |
| 8046/3: Non-small cell carcinoma | 0 | 0 | 9 | 1 | 17 | 27 (0.58%) |
| 8050/3: Papillary carcinoma, NOS | 0 | 0 | 0 | 0 | 1 | 1 (0.02%) |
| 8051/3: Verrucous carcinoma, NOS | 0 | 0 | 0 | 0 | 1 | 1 (0.02%) |
| 8052/3: Papillary squamous cell carcinoma | 2 | 3 | 3 | 0 | 11 | 19 (0.41%) |
| 8070/3: Squamous cell carcinoma, NOS | 33 | 238 | 576 | 69 | 402 | 1318 (28.30%) |
| 8071/3: Squamous cell carcinoma, keratinizing, NOS | 11 | 66 | 55 | 5 | 47 | 184 (3.95%) |
| 8072/3: Squamous cell carcinoma, large cell, nonkeratinizing, NOS | 13 | 47 | 329 | 199 | 284 | 872 (18.72%) |
| 8073/3: Squamous cell carcinoma, small cell, nonkeratinizing | 0 | 2 | 11 | 14 | 4 | 31 (0.67%) |
| 8074/3: Squamous cell carcinoma, spindle cell | 0 | 0 | 8 | 1 | 8 | 17 (0.36%) |
| 8075/3: Squamous cell carcinoma, adenoid | 0 | 0 | 1 | 0 | 0 | 1 (0.02%) |
| 8082/3: Lymphoepithelial carcinoma | 0 | 1 | 43 | 193 | 66 | 303 (6.50%) |
| 8083/3: Basaloid squamous cell carcinoma | 0 | 1 | 19 | 1 | 17 | 38 (0.82%) |
| 8084/3: Squamous cell carcinoma, clear cell type | 0 | 0 | 0 | 0 | 1 | 1 (0.02%) |
| 8090/3: Basal cell carcinoma, NOS | 0 | 0 | 1 | 0 | 0 | 1 (0.02%) |
| 8120/3: Transitional cell carcinoma, NOS | 0 | 0 | 0 | 0 | 1 | 1 (0.02%) |
| 8123/3: Basaloid carcinoma | 0 | 0 | 2 | 1 | 4 | 7 (0.15%) |
| 8140/3: Adenocarcinoma, NOS | 3 | 6 | 8 | 1 | 5 | 23 (0.49%) |
| 8145/3: Carcinoma, diffuse type | 0 | 0 | 0 | 1 | 1 | 2 (0.04%) |
| 8200/3: Adenoid cystic carcinoma | 3 | 2 | 2 | 1 | 32 | 40 (0.86%) |
| 8211/3: Tubular adenocarcinoma | 0 | 0 | 0 | 0 | 1 | 1 (0.02%) |
| 8240/3: Carcinoid tumor, NOS | 0 | 0 | 0 | 1 | 0 | 1 (0.02%) |
| 8246/3: Neuroendocrine carcinoma, NOS | 1 | 0 | 5 | 5 | 3 | 14 (0.30%) |
| 8260/3: Papillary adenocarcinoma, NOS | 1 | 4 | 0 | 0 | 1 | 6 (0.13%) |
| 8310/3: Clear cell adenocarcinoma, NOS | 0 | 0 | 0 | 0 | 3 | 3 (0.06%) |
| 8430/3: Mucoepidermoid carcinoma | 3 | 3 | 2 | 4 | 2 | 14 (0.30%) |
| 8440/3: Cystadenocarcinoma, NOS | 0 | 0 | 0 | 0 | 1 | 1 (0.02%) |
| 8450/3: Papillary cystadenocarcinoma, NOS | 0 | 1 | 0 | 0 | 0 | 1 (0.02%) |
| 8525/3: Polymorphous low grade adenocarcinoma | 1 | 5 | 0 | 0 | 0 | 6 (0.13%) |
| 8560/3: Adenosquamous carcinoma | 0 | 0 | 3 | 0 | 3 | 6 (0.13%) |
| 8562/3: Epithelial-myoepithelial carcinoma | 1 | 3 | 0 | 0 | 1 | 5 (0.11%) |
| 8574/3: Adenocarcinoma with neuroendocrine differentiation | 0 | 0 | 1 | 0 | 1 | 2 (0.04%) |
| 8720/3: Malignant melanoma, NOS | 0 | 0 | 0 | 0 | 6 | 6 (0.13%) |
| 8746/3: Mucosal lentiginous melanoma | 0 | 0 | 0 | 0 | 1 | 1 (0.02%) |
| 8772/3: Spindle cell melanoma, NOS | 0 | 0 | 0 | 1 | 0 | 1 (0.02%) |
| 8800/3: Sarcoma, NOS | 0 | 0 | 0 | 1 | 2 | 3 (0.06%) |
| 8801/3: Spindle cell sarcoma | 0 | 2 | 0 | 0 | 0 | 2 (0.04%) |
| 8890/3: Leiomyosarcoma, NOS | 0 | 0 | 1 | 0 | 0 | 1 (0.02%) |
| 8900/3: Rhabdomyosarcoma, NOS | 0 | 0 | 3 | 0 | 3 | 6 (0.13%) |
| 8902/3: Mixed type rhabdomyosarcoma | 0 | 0 | 0 | 0 | 1 | 1 (0.02%) |
| 8910/3: Embryonal rhabdomyosarcoma, NOS | 0 | 0 | 1 | 2 | 26 | 29 (0.62%) |
| 8920/3: Alveolar rhabdomyosarcoma | 0 | 1 | 0 | 1 | 11 | 13 (0.28%) |
| 8941/3: Carcinoma in pleomorphic adenoma | 0 | 0 | 1 | 0 | 1 | 2 (0.04%) |
| 8980/3: Carcinosarcoma, NOS | 0 | 0 | 0 | 1 | 0 | 1 (0.02%) |
| 8982/3: Malignant myoepithelioma | 0 | 0 | 1 | 0 | 0 | 1 (0.02%) |
| 9040/3: Synovial sarcoma, NOS | 0 | 1 | 0 | 0 | 0 | 1 (0.02%) |
| 9071/3: Yolk sac tumor | 0 | 0 | 0 | 0 | 2 | 2 (0.04%) |
| 9150/3: Hemangiopericytoma, malignant | 0 | 0 | 0 | 0 | 2 | 2 (0.04%) |
| 9220/3: Chondrosarcoma, NOS | 1 | 0 | 0 | 0 | 0 | 1 (0.02%) |
| 9231/3: Myxoid chondrosarcoma | 0 | 1 | 0 | 0 | 0 | 1 (0.02%) |
| 9370/3: Chordoma, NOS | 0 | 0 | 0 | 0 | 5 | 5 (0.11%) |
| 9371/3: Chondroid chordoma | 0 | 1 | 0 | 0 | 1 | 2 (0.04%) |
| 9500/3: Neuroblastoma, NOS | 0 | 1 | 0 | 0 | 0 | 1 (0.02%) |
| 9522/3: Olfactory neuroblastoma | 0 | 0 | 2 | 0 | 2 | 4 (0.09%) |
| 9540/3: Malignant peripheral nerve sheath tumor | 0 | 0 | 0 | 0 | 1 | 1 (0.02%) |
| Total | 78 | 395 | 1381 | 1288 | 1516 | 4658 (100.00%) |
