# Supplementary material for: Benefits of local tumor excision and pharyngectomy on the survival of nasopharyngeal carcinoma patients: a retrospective observational study based on SEER database
Source: J Transl Med. 2017 May 30;15:116. doi: 10.1186/s12967-017-1204-x (PMC5450381; doi:10.1186/s12967-017-1204-x)
Supplement: Supplementary file 2 — Additional file 2: Figure S1. ROC for Age (AUC = 0.6423, P < 0.0001): outcome is all cause mortality (n = 4658). Table S2. 60 year is the optimal cutoff for age as a predictor of all-cause mortality. Figure S2. ROC for Age (AUC = 0.6423, P < 0.0001): outcome is cancer-specific mortality (n = 3894). Table S3. 60 year is the optimal cutoff for age as a predictor of all-cause mortality. [file 12967_2017_1204_MOESM2_ESM.docx]

**12967_2017_1204_MOESM2_ESM**

**Figure S1. ROC for Age (AUC=0.6423, P<0.0001): outcome is all cause mortality (n=4,658).**

**Table S2. 60yr is the optimal cutoff for age as a predictor of all-cause mortality**

**Figure S2. ROC for Age (AUC=0.6423, P<0.0001): outcome is cancer-specific mortality (n=3,894).**

**Table S3. 60yr is the optimal cutoff for age as a predictor of all-cause mortality**

**Figure S1.**


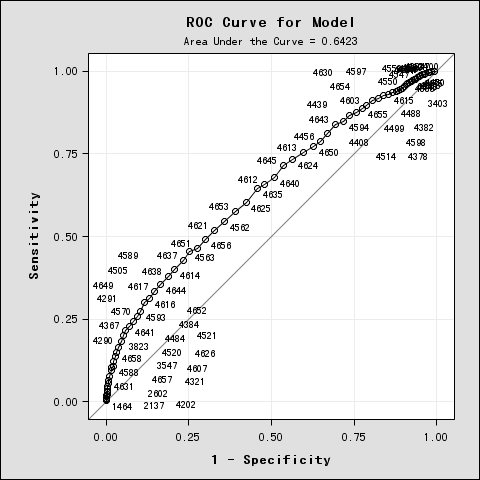


**Table S2.**

| ID in the graph | Age | Sensitivity | Specificity |
| --- | --- | --- | --- |
| 4,656 | 58 | 0.53283 | 0.70021 |
| 4,651 | 59 | 0.50576 | 0.72519 |
| 4,579 | 60 | 0.48675 | 0.74778 |
| 4,637 | 61 | 0.46544 | 0.76728 |
| 4,614 | 62 | 0.43952 | 0.79295 |

**Figure S2.**


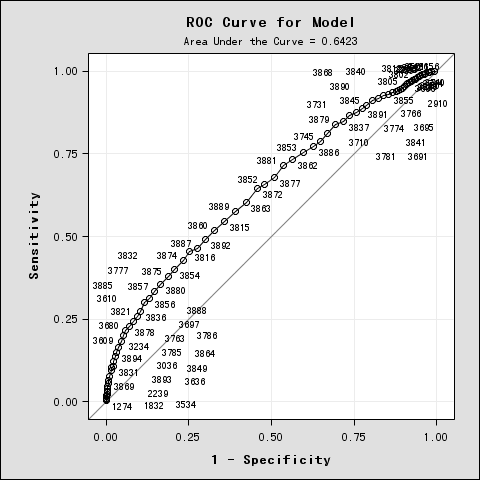


**Table S3.**

| ID in the graph | Age | Sensitivity | Specificity |
| --- | --- | --- | --- |
| 3,892 | 58 | 0.48971 | 0.70021 |
| 3,887 | 59 | 0.46502 | 0.72519 |
| 3,816 | 60 | 0.45473 | 0.74778 |
| 3,874 | 61 | 0.42798 | 0.76728 |
| 3,854 | 62 | 0.40123 | 0.79295 |
