# Supplementary material for: Benefits of local tumor excision and pharyngectomy on the survival of nasopharyngeal carcinoma patients: a retrospective observational study based on SEER database
Source: J Transl Med. 2017 May 30;15:116. doi: 10.1186/s12967-017-1204-x (PMC5450381; doi:10.1186/s12967-017-1204-x)
Supplement: Supplementary file 3 — Additional file 3: Figure S3. Kaplan Meier Curve for all-cause mortality (A) and nasopharyngeal carcinoma-specific mortality (B). Table S4. Survival rate information for all cause mortality. Table S5. Survival rate information for nasopharyngeal carcinoma-specific mortality. [file 12967_2017_1204_MOESM3_ESM.docx]

**12967_2017_1204_MOESM3_ESM**

**Figure S3. Kaplan Meier Curve for all-cause mortality (A) and nasopharyngeal carcinoma-specific mortality (B).**

**Table S4. Survival rate information for all cause mortality.**

**Table S5. Survival rate information for nasopharyngeal carcinoma-specific mortality.**

**Figure S3.**

1. **B.**
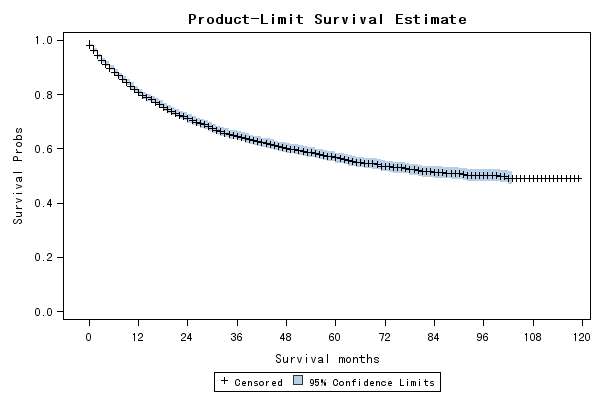

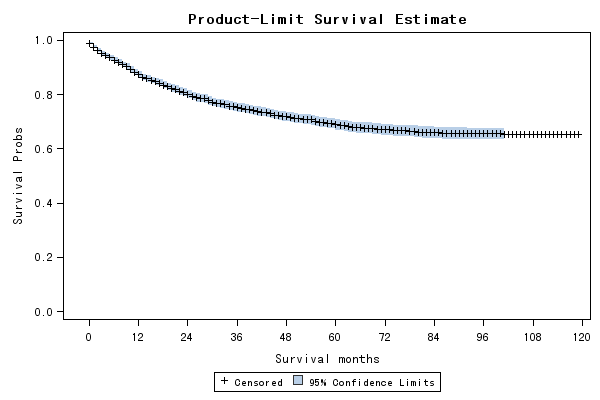


**Table S4.**

| Timelist (months) | Months | Survival | Number Failed | Number Left |
| --- | --- | --- | --- | --- |
| 12 | 12 | 0.8068 | 708 | 2702 |
| 24 | 24 | 0.7105 | 1012 | 2068 |
| 36 | 36 | 0.6469 | 1186 | 1605 |
| 48 | 48 | 0.6021 | 1289 | 1248 |
| 60 | 60 | 0.5667 | 1355 | 930 |
| 72 | 72 | 0.5351 | 1401 | 655 |
| 84 | 84 | 0.5135 | 1424 | 457 |
| 96 | 96 | 0.5013 | 1433 | 285 |
| 108 | 103 | 0.4906 | 1438 | 135 |
| 120 | 103 |  | 1438 | 0 |

**Table S5.**

| Timelist (months) | Months | Survival | Number Failed | Number Left |
| --- | --- | --- | --- | --- |
| 12 | 12 | 0.8730 | 386 | 2403 |
| 24 | 24 | 0.8016 | 570 | 1889 |
| 36 | 36 | 0.7523 | 679 | 1491 |
| 48 | 48 | 0.7188 | 740 | 1176 |
| 60 | 60 | 0.6888 | 784 | 880 |
| 72 | 71 | 0.6713 | 804 | 631 |
| 84 | 84 | 0.6591 | 814 | 446 |
| 96 | 92 | 0.6557 | 816 | 281 |
| 108 | 101 | 0.6529 | 817 | 135 |
| 120 | 101 |  | 817 | 0 |
