# Supplementary material for: Benefits of local tumor excision and pharyngectomy on the survival of nasopharyngeal carcinoma patients: a retrospective observational study based on SEER database
Source: J Transl Med. 2017 May 30;15:116. doi: 10.1186/s12967-017-1204-x (PMC5450381; doi:10.1186/s12967-017-1204-x)

**12967_2017_1204_MOESM4_ESM**

**Figure S4. Comparison of standardized differences (A, B) and propensity scores (C, D) in unmatched and matched samples. A, C: propensity score matching in overall dataset; B, D: propensity score matching of dataset excluding deaths with other reasons.**

**
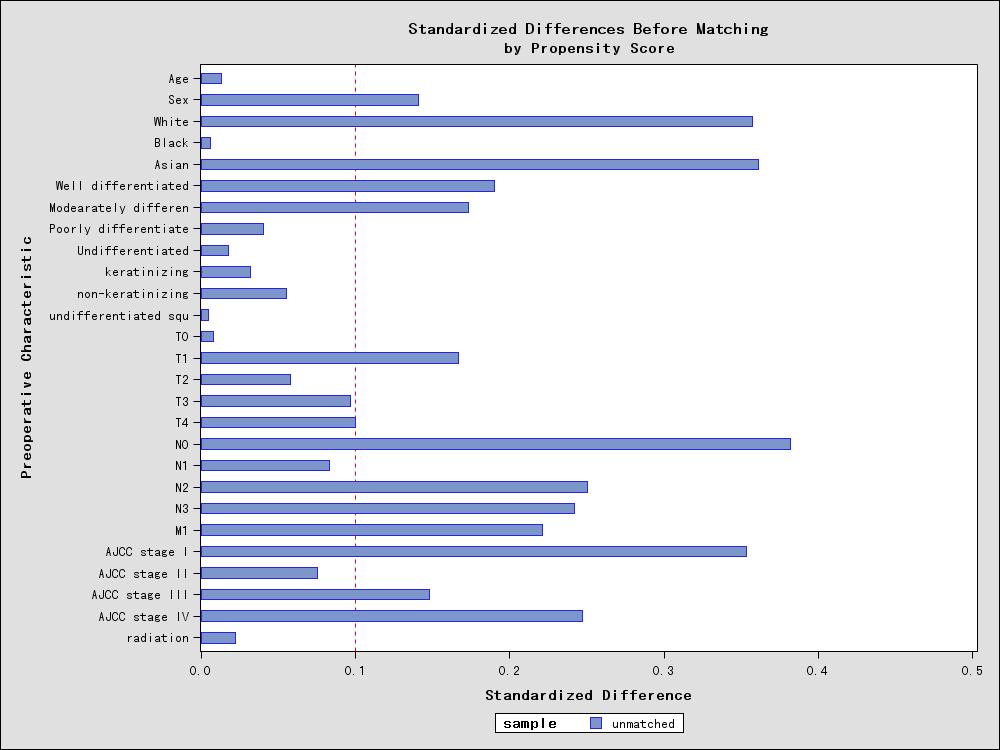

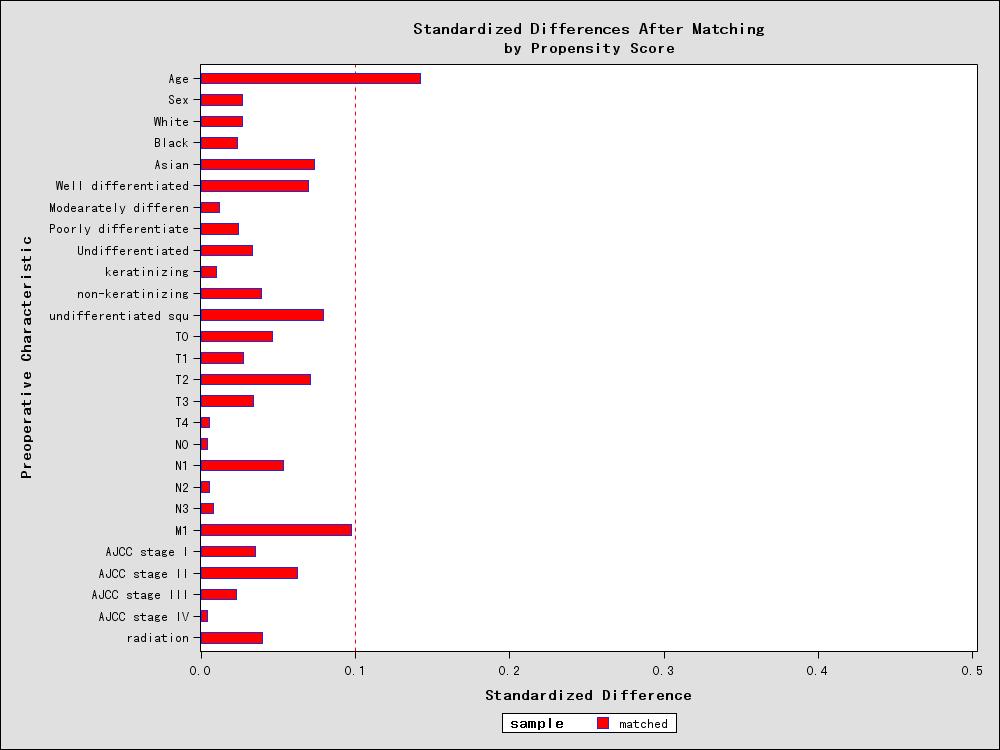
**

B.


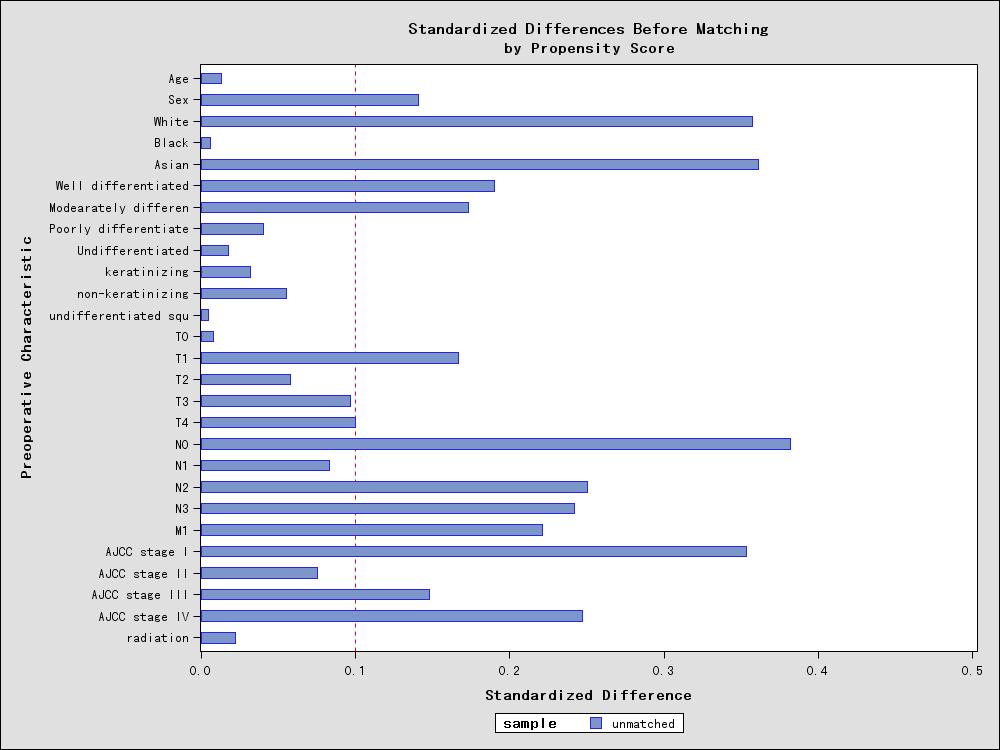

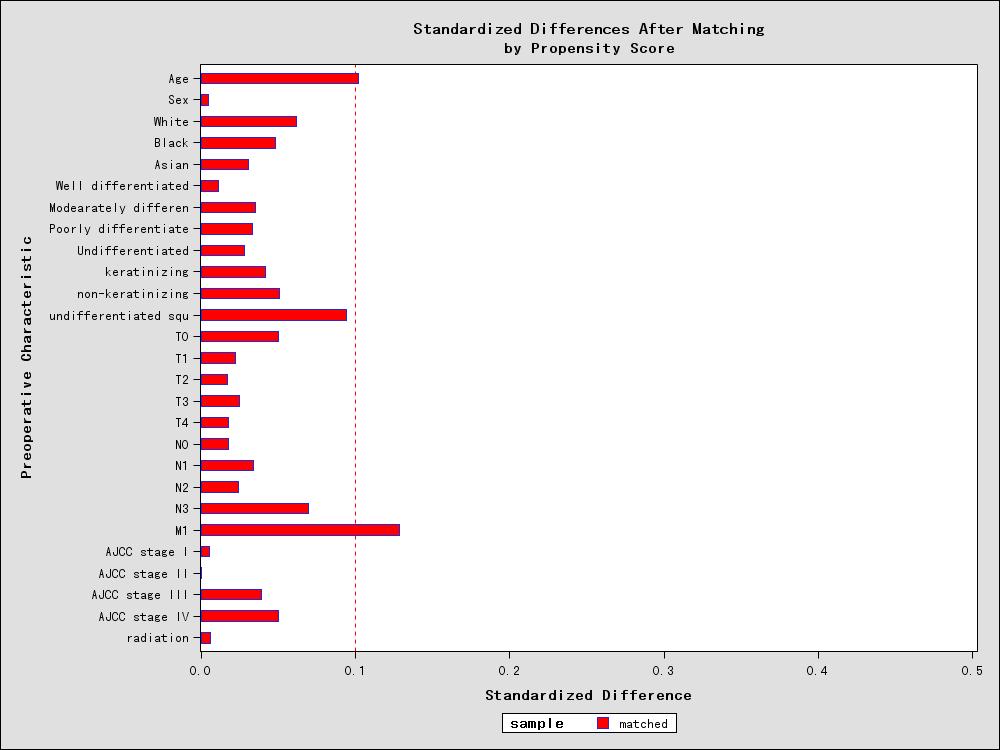


**C.**   **D.**


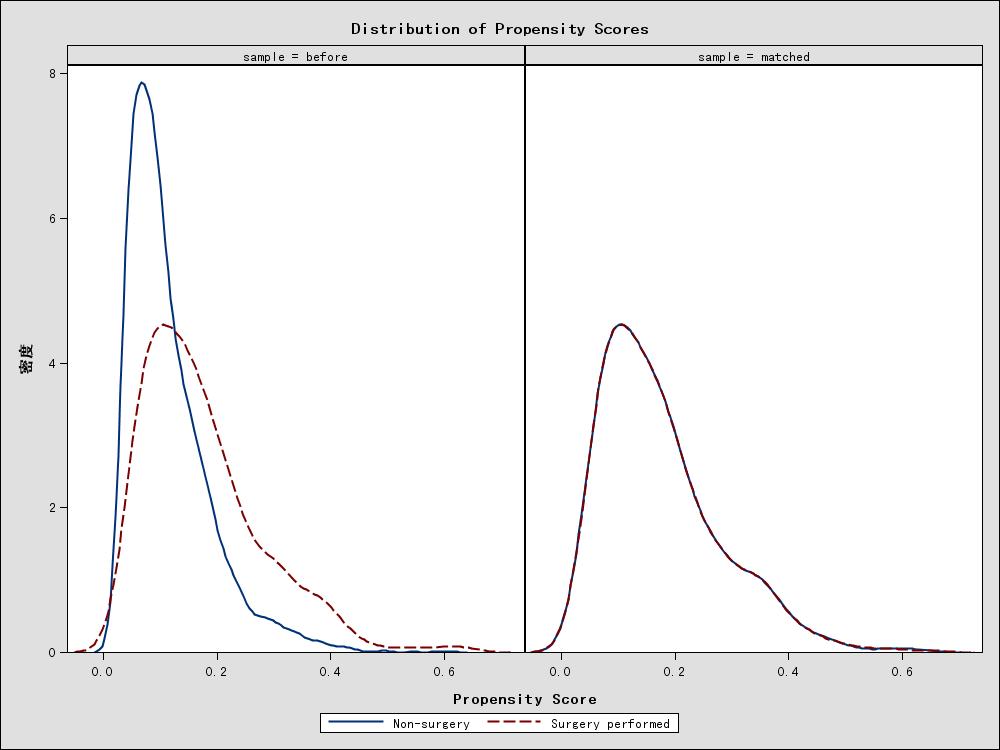

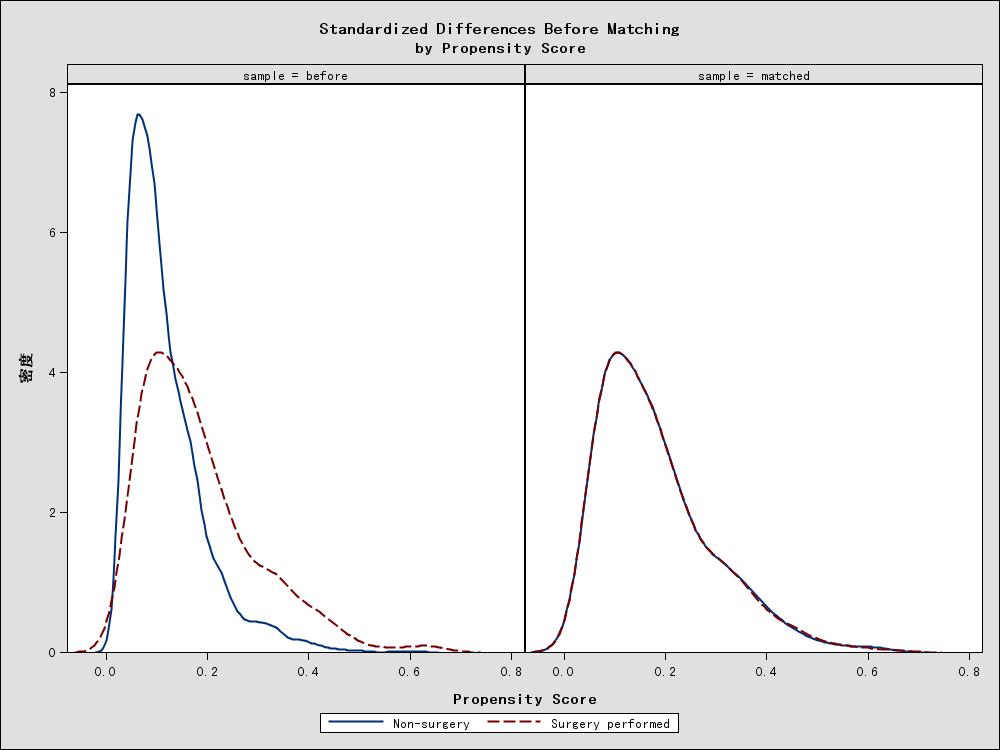

Supplement: Supplementary file 4 — Additional file 4: Figure S4. Comparison of standardized differences (A, B) and propensity scores (C, D) in unmatched and matched samples. A, C: propensity score matching in overall dataset; B, D: propensity score matching of dataset excluding deaths with other reasons. [file 12967_2017_1204_MOESM4_ESM.docx]
