# Supplementary material for: Benefits of local tumor excision and pharyngectomy on the survival of nasopharyngeal carcinoma patients: a retrospective observational study based on SEER database
Source: J Transl Med. 2017 May 30;15:116. doi: 10.1186/s12967-017-1204-x (PMC5450381; doi:10.1186/s12967-017-1204-x)
Supplement: Supplementary file 5 — Additional file 5: Figure S5. Kaplan Meier Curve for all-cause mortality (A) and nasopharyngeal carcinoma-specific mortality (B) stratified by surgery. Table S6. Survival rate information for all cause mortality stratified by surgery. Table S7. Survival rate information for nasopharyngeal carcinoma-specific mortality stratified by surgery. [file 12967_2017_1204_MOESM5_ESM.docx]

**12967_2017_1204_MOESM5_ESM**

**Figure S5. Kaplan Meier Curve for all-cause mortality (A) and nasopharyngeal carcinoma-specific mortality (B) stratified by surgery.**

**Table S6. Survival rate information for all cause mortality stratified by surgery.**

**Table S7. Survival rate information for nasopharyngeal carcinoma-specific mortality stratified by surgery.**

**Figure S5.**

1. B.


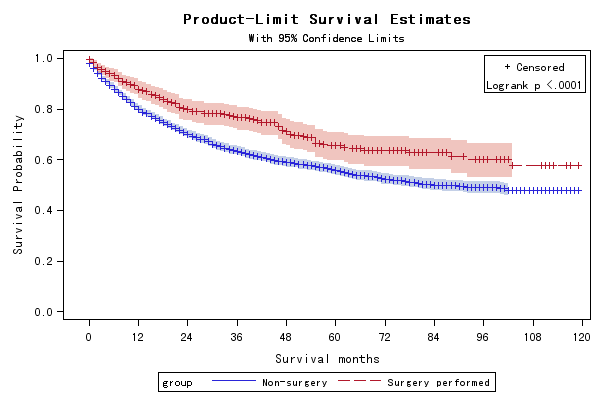

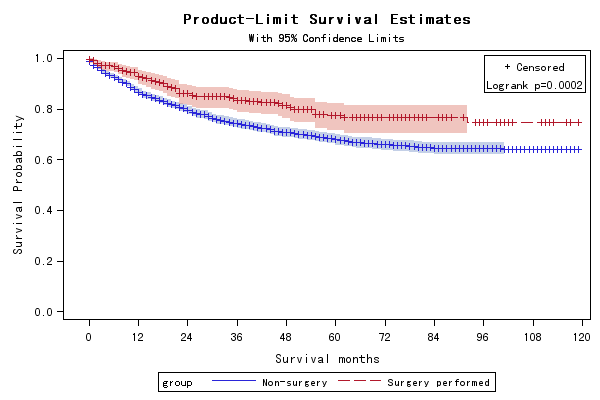


**Table S6.**

|  | Non-surgery | | | | Surgery | | | |
| --- | --- | --- | --- | --- | --- | --- | --- | --- |
| Timelist (months) | Event Months | Survival probability | Number Failed | Number Left | Event Months | Survival probability | Number Failed | Number Left |
| 12 | 12 | 0.7990 | 654 | 2369 | 12 | 0.8787 | 46 | 313 |
| 24 | 24 | 0.7003 | 930 | 1798 | 24 | 0.7985 | 73 | 253 |
| 36 | 36 | 0.6336 | 1091 | 1390 | 36 | 0.7646 | 83 | 204 |
| 48 | 48 | 0.5900 | 1180 | 1082 | 48 | 0.7100 | 96 | 158 |
| 60 | 60 | 0.5567 | 1235 | 811 | 58 | 0.6547 | 107 | 112 |
| 72 | 72 | 0.5232 | 1278 | 566 | 67 | 0.6357 | 110 | 82 |
| 84 | 84 | 0.5000 | 1300 | 395 | 78 | 0.6266 | 111 | 57 |
| 96 | 96 | 0.4892 | 1307 | 245 | 92 | 0.6009 | 113 | 36 |
| 108 | 102 | 0.4796 | 1311 | 114 | 103 | 0.5769 | 114 | 20 |
| 120 | 102 |  | 1311 | 0 | 103 |  | 114 | 0 |

**Table S7.**

|  | Non-surgery | | | | Surgery | | | |
| --- | --- | --- | --- | --- | --- | --- | --- | --- |
| Timelist (months) | Event months | Survival probability | Number Failed | Number Left | Event months | Survival probability | Number Failed | Number Left |
| 12 | 12 | 0.8668 | 358 | 2102 | 12 | 0.9294 | 23 | 284 |
| 24 | 24 | 0.7946 | 522 | 1643 | 24 | 0.8595 | 43 | 231 |
| 36 | 36 | 0.7425 | 623 | 1295 | 36 | 0.8354 | 49 | 186 |
| 48 | 48 | 0.7070 | 680 | 1019 | 47 | 0.8154 | 53 | 149 |
| 60 | 60 | 0.6783 | 717 | 766 | 58 | 0.7728 | 60 | 107 |
| 72 | 71 | 0.6595 | 736 | 545 | 62 | 0.7654 | 61 | 79 |
| 84 | 84 | 0.6457 | 746 | 386 | 62 | 0.7654 | 61 | 55 |
| 96 | 86 | 0.6440 | 747 | 242 | 92 | 0.7654 | 62 | 35 |
| 108 | 101 | 0.6408 | 748 | 114 | 92 | 0.7654 | 62 | 20 |
| 120 | 101 |  | 748 | 0 | 92 |  | 62 | 0 |
